# Lantern: an integrative repository of functional annotations for lncRNAs in the human genome

**DOI:** 10.1186/s12859-021-04207-3

**Published:** 2021-05-26

**Authors:** Swapna Vidhur Daulatabad, Rajneesh Srivastava, Sarath Chandra Janga

**Affiliations:** 1 Department of BioHealth Informatics, School of Informatics and Computing, Indiana University Purdue University, Informatics and Communications Technology Complex, 535 W Michigan St., IT 475H, Indianapolis, IN 46202 USA; 2 Department of Surgery, Indiana Center for Regenerative Medicine and Engineering (ICRME), Indiana University School of Medicine, Indianapolis, IN 46202 USA; 3 Department of Medical and Molecular Genetics, Medical Research and Library Building, Indiana University School of Medicine, 975 West Walnut Street, Indianapolis, IN 46202 USA; 4 Centre for Computational Biology and Bioinformatics, Indiana University School of Medicine, 5021 Health Information and Translational Sciences (HITS), 410 West 10th Street, Indianapolis, IN 46202 USA

**Keywords:** LncRNA, Ontology, Expression, Interaction, eCLIP, Coding potential, Snps, eQTL

## Abstract

**Background:**

With advancements in omics technologies, the range of biological processes where long non-coding RNAs (lncRNAs) are involved, is expanding extensively, thereby generating the need to develop lncRNA annotation resources. Although, there are a plethora of resources for annotating genes, despite the extensive corpus of lncRNA literature, the available resources with lncRNA ontology annotations are rare.

**Results:**

We present a lncRNA annotation extractor and repository (Lantern), developed using PubMed’s abstract retrieval engine and NCBO’s recommender annotation system. Lantern’s annotations were benchmarked against lncRNAdb’s manually curated free text. Benchmarking analysis suggested that Lantern has a recall of 0.62 against lncRNAdb for 182 lncRNAs and precision of 0.8. Additionally, we also annotated lncRNAs with multiple omics annotations, including predicted cis-regulatory TFs, interactions with RBPs, tissue-specific expression profiles, protein co-expression networks, coding potential, sub-cellular localization, and SNPs for ~ 11,000 lncRNAs in the human genome, providing a one-stop dynamic visualization platform.

**Conclusions:**

Lantern integrates a novel, accurate semi-automatic ontology annotation engine derived annotations combined with a variety of multi-omics annotations for lncRNAs, to provide a central web resource for dissecting the functional dynamics of long non-coding RNAs and to facilitate future hypothesis-driven experiments. The annotation pipeline and a web resource with current annotations for human lncRNAs are freely available on sysbio.lab.iupui.edu/lantern.

**Supplementary Information:**

The online version contains supplementary material available at 10.1186/s12859-021-04207-3.

## Background

Non-coding RNAs across the genome have been associated with a variety of biological processes [1–4], ranging from regulation of splicing to remodeling of chromatin [5, 6]. Amongst the repertoire of non-coding sequences, lies a critical species of RNAs called long non-coding RNAs (lncRNAs) [7]. An increasing number of studies suggest that lncRNAs significantly contribute to a large spectrum of human phenotypes including cancers [8–10], neurological disorders such as Alzheimer’s disease [11], Heart failure [12], and Diabetes [13]. However, the complete functional role of lncRNAs is not fully characterized [14–17]. Hence, uncovering the role of lncRNAs in disease phenotypes is critical for targeting them as potential therapeutic agents [18, 19].

This rapidly emerging field, therefore, requires the development of a robust framework for comprehensive annotations of lncRNAs. Several approaches have been published so far to annotate and characterize lncRNAs [20–23]. The GENCODE project [24] has identified thousands of lncRNAs across the human genome out of which only a small percentage are functionally understood, leaving a significant part of the functional non-coding genome unexplored.

Currently, available lncRNA annotation resources attempt to bridge the gap between lncRNAs and their biological functions. Although resources like Noncode [25], lncRNAdb [26], lncRNome [27], LNCipedia [28] and lncRNADisease [29] have functionally annotated lncRNAs, they are either limited to a small set of lncRNAs or do not provide an extensive understanding of their biological functions.

In addition to controlled annotations, the available resources have not elaborated other key parameters that elucidate the role of lncRNAs like their tissue specificity, subcellular compartment localization, and cross-talk with various cellular components.

In this study, we propose a lncRNA annotation extractor and repository, Lantern, which renders users annotate lncRNA with multiple ontologies along with multi-omic lncRNA annotations. Lantern integrates lncRNA functional ontology information extracted from natural language processing of abstracts from PubMed, protein -RNA interactions obtained from CLIP experiments, expression profiles across human tissues, and lncRNA characteristics like protein-coding potential, SNPs, and sub-cellular localization into one resource.

Availability of such functional annotations for lncRNAs can aid experimental scientists to design hypothesis-driven research based on annotations from Lantern. Therefore, Lantern will significantly aid our understanding of the non-coding transcriptome and its role in human disease phenotypes.

### Overview of Lantern

**Fig. 1:**
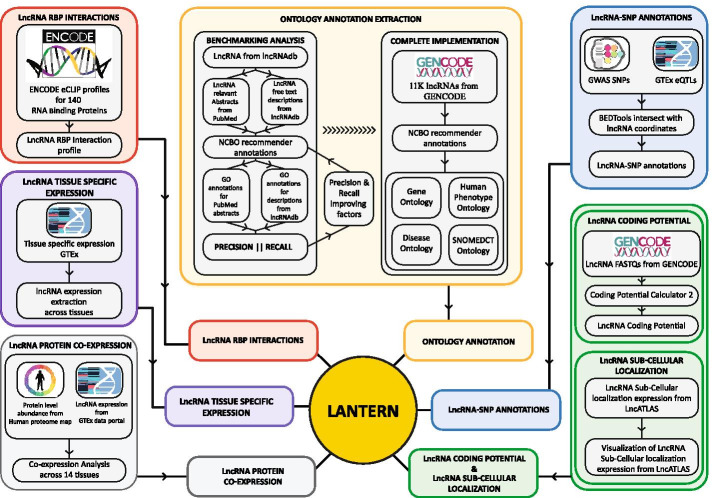
Lantern workflow overview. The flow chart shows various NGS and ontology extraction pipelines merging to annotate lncRNAs with multi-level omics and ontology information. All the annotation pipelines were integrated, and the extracted annotations are hosted on the online resource. The ontology annotation extraction pipeline showing a two-part workflow, the first part benchmarks the pipeline developed, and the annotations extracted, second part shows the implementation of the developed pipeline over all the human lncRNAs from GENCODE. Respective flowcharts on either side showing data resources and steps involved in extracting a diverse set of omics data for annotating lncRNAs

Lantern hosts multi-omic annotation information for 11,290 lncRNAs, amongst which, 769 lncRNAs are annotated with multiple functional ontology information extracted from 6942 abstracts of lncRNA pertaining literature. A total of 9982 lncRNAs were annotated with tissue-specific expression across 53 tissues from the GTEx data portal, and 6714 lncRNAs were annotated with SNPs across 48 tissues based on eQTL and SNP information from GTEx and GWAS studies. Additionally, the protein-coding potential of 9898 lncRNAs was computed and recorded. Sub-cellular localization of 11,290 lncRNAs in 10 cellular compartments across 15 cell lines was also obtained and hosted on Lantern. A total of 617,074 interactions across 161 transcription factors and 10,727 lncRNAs were also predicted and mounted onto the Lantern interface. RNA binding protein (RBP) interactions of 7942 lncRNAs across 18 cell lines were computed from ENCODE eCLIP data, along with protein co-expression information, extracted from analysis based on information from Human Proteome Map and GTEx, for 5331 lncRNAs are available on Lantern (see Construction and Content section). Table 1 summarizes the various datasets employed in constructing Lantern. The functional ontology annotations extracted by the NLP-based literature mining framework, the Mobius pipeline, were benchmarked against the manually curated gold standard annotations from lncRNAdb. From the benchmarking analysis, we observe that our pipeline can recall 62% of the original annotations, and the extracted annotations had a precision of 80% (see “Construction and Content” section). Around 26% of all the lncRNAs with gene ontology annotations extracted using our novel Mobius pipeline could be benchmarked based on available annotations from prior resources. Across these Mobius pipeline-based annotations of lncRNAs, we observed that ‘NEOPLASM’/‘TUMOR’ (292 lncRNAs associated), ‘TYPE I PROGRAMMED CELL DEATH’ (240 lncRNAs associated) ontologies were associated with the most number of lncRNAs. Users can search and visualize various levels of lncRNA annotations, information including where a lncRNA localizes in the cell, which tissue is more representative of a specific lncRNA, which RBPs interact with which lncRNAs, SNPs prevalent on lncRNA specific to tissue and phenotype, and most importantly controlled ontology annotations. Lantern enables users to understand the interactive nature of lncRNAs and visualize characteristics of lncRNAs to design studies that help further explore the functional scope of the non-coding transcriptome. As conceptualized in Fig. 1, Lantern is a resource generated by the integration of multiple pipelines extracting and presenting several key levels of lncRNA annotation information.

**Table 1:** Various annotation components available on Lantern for lncRNAs along with the number of annotated lncRNAs with respective annotations along with their coverage

Annotation	Number of lncRNAs with respective annotation	Features
Ontology	769	Gene ontology, human phenotype ontology, disease ontology, and SNOMEDCT ontology extracted from 6942 abstracts
RBP-interactions	7942	Across 18 cell lines
Tissue-specific expression	9982	Across 53 tissues
Protein co-expression	5331	Across 14 tissues
Transcription factor interaction	10,727	Potential 617,074 regulatory interactions with 161 Transcription Factors
Coding Potential	9898	For 27,907 transcripts
Sub-cellular localization	11,290	For 10 cellular compartments across 15 cell lines
GTEx eQTL SNPs	6714	Across 48 tissues
GWAS SNPs	2569	1421 phenotypic characteristics

### Construction and content

In this study, we integrated multiple pipelines to extract functional annotations and cellular interactions of lncRNAs as illustrated in Fig. 1. The crux of Lantern is a semi-automated ontology annotation pipeline, hereby referred to as the “Mobius” pipeline, which uses concepts of Natural Language Processing (NLP) to mine lncRNA relevant literature to extract accurate non-coding transcriptome ontology annotations. The Mobius pipeline annotates lncRNAs with 4 ontologies: gene ontology (GO), human phenotype ontology (HPO), disease ontology (DO), and SNOMEDCT ontology. The annotations extracted by the Mobius pipeline were benchmarked against an established gold standard repository, and the pipeline was deployed to overall GENCODE registered lncRNAs and the data was deposited onto an online resource. Additionally, we used established Next-Generation Sequencing (NGS) data analysis approaches to annotate lncRNAs with diverse omics information such as lncRNA-RBP interactions, tissue-specific lncRNA expression, lncRNA-protein co-expression, coding potential, sub-cellular localization, and SNPs in lncRNA.

### Ontology annotation for long non-coding RNAs (lncRNAs)

The lncRNA ontology annotation extraction pipeline, the Mobius pipeline, contains two parts as seen in Fig. 1. In the first part of this implementation, the pipeline is deployed over a subset of lncRNAs and benchmarked against an established manually curated lncRNA annotation resource. In the second part, the benchmarked pipeline is deployed over human genome-wide lncRNAs from GENCODE, to extract annotations for all the lncRNAs that have been studied so far. The individual steps involved in the Mobius pipeline are as follows:

#### Abstract extraction for lncRNA relevant literature from PubMed

Our gold standard for this study, lncRNAdb [26] annotates lncRNAs with function, localization, characteristics, and conservation information by manual curation of literature and recording information for each lncRNA, thereby making it an appropriate gold standard. We extracted all the 298 lncRNA names from lncRNAdb which had characteristic information. These extracted lncRNA names were then plugged into PubMed’s search engine via an application programming interface (API) using python, to retrieve abstracts relevant to the respective lncRNA. To make the results precise, additional key terms such as ‘RNA’, ‘lncRNA’, ‘long non-coding RNA’, and ‘non-coding RNA’ were added to the lncRNA names before performing the search. The resulting hits were obtained as XML of PMIDs. These PMIDs were then used to obtain the abstracts using PubMed eUtils [30]. The output abstracts were also extracted in XML and were parsed using python to extract specific information like abstract, and PMID.

#### Ontology extraction using NCBO recommender system

The National Center for Biomedical Ontology (NCBO) [31] recommender parses a given input text to identify and suggest the most representative ontology based on the keywords contained in the text. To identify the functional ontology terms, the extracted abstracts were pushed to the NCBO ontology recommender endpoint using REST API [32]. The NCBO ontology recommender then identified and returned the annotations in the posted text with respect to 4 ontologies; Gene ontology [33], Human phenotype ontology [34], Disease ontology [35], and SNOMEDCT [36]. The returned ontology associations were annotated with the respective lncRNAs. Along with lncRNA-specific ontology annotation, the corresponding ontology IDs were extracted and recorded.

#### Benchmarking predicted lncRNA ontology annotations against a gold standard

To measure the accuracy of the extracted ontology information, we compared our annotations with those from lncRNAdb, across 182 lncRNAs that had annotation from both sources. We extracted all the characteristic descriptions of each lncRNA from lncRNAdb as free text. The extracted free text was then parsed through NCBO ontology recommender as previously performed with lncRNA abstracts from PubMed. Thereby obtaining annotations for gold-standard information, which was now comparable to the annotations extracted from PubMed abstracts. The number of ontology IDs common across annotations from the Mobius pipeline and gold standard free text from lncRNAdb was computed for each lncRNA, based on which the recall was calculated using the following equation (1).

1 $${\text{Recall}} = \frac{{{\text{Number}}\,{\text{of}}\,{\text{shared}}\,{\text{annotations}}\,{\text{across}}\,{\text{extracted}}\,{\text{and}}\,{\text{gold}}\,{\text{standard}}\,{\text{annotations}}}}{{{\text{Total}}\,{\text{number}}\,{\text{of}}\,{\text{annotations}}\,{\text{extracted}}}}$$

The lncRNA functional ontology annotations extracted by the Mobius pipeline demonstrated a recall of 62% across the annotations extracted from the gold standard, lncRNAdb.

Since the gold standard lacks updated annotations and the Mobius pipeline extracts annotations from the literature until December 2018, precision was calculated in a non-traditional approach. Precision was manually computed by randomly selecting 50 lncRNAs, reading through each of the annotated abstracts to identify which annotations from the Mobius pipeline were accurate and which were misannotated. Subsequently, the precision of the Mobius pipeline was computed as 80% using equation (2). The record of this curation step is provided in Additional file 1: Table S1.

2 $${\text{Precision}} = \frac{{{\text{Number}}\,{\text{of}}\,{\text{true}}\,{\text{annotations}}\,{\text{extracted}}}}{{{\text{Total}}\,{\text{number}}\,{\text{of}}\,{\text{annotations }}\,{\text{extracted}}}}$$

#### Genome-scale ontology annotation for lncRNAs using Mobius pipeline

As seen in Fig. 1, after the benchmarking analysis, the Mobius pipeline was deployed overall 11,405 human lncRNAs recorded in GENCODE, to annotate the non-coding transcriptome as much as possible with functional ontology terms. The PubMed abstract retrieval system was employed over these 11,405 lncRNAs, out of which 769 lncRNAs had at least one abstract mentioning the respective lncRNA’s name in the abstract or title of the article. A total of 6942 unique abstracts were extracted for 769 lncRNAs. These abstracts were then parsed by the NCBO ontology recommender to mine ontology annotations present in the free text. The PMIDs and their respective ontology annotations were retrieved and reannotated to the respective lncRNAs, to obtain 698 lncRNAs with Gene Ontology annotations, 433 lncRNAs with Human Phenotype ontology annotations, 239 lncRNAs with Disease ontology annotations and 357 lncRNAs with SNOMEDCT ontology annotations. These annotations were formatted into tables and fed into the database to be visualized as interaction network of lncRNAs and respective ontology annotations on Lantern, the same information can also be visualized in form of tables.

**Fig. 2:**
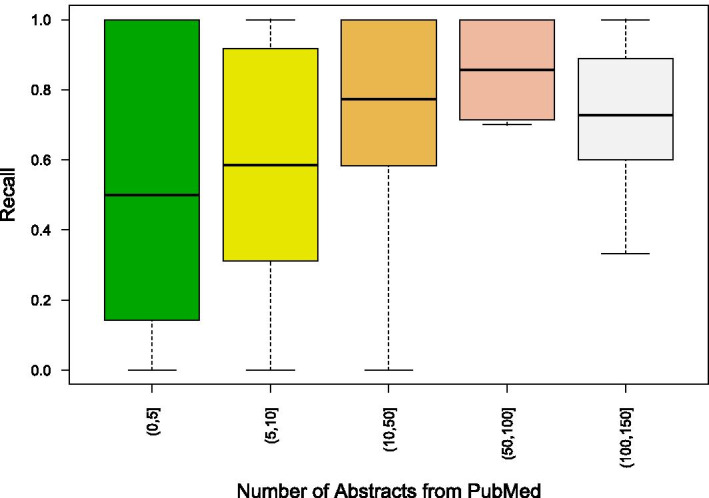
Boxplots showing the distribution of recall values across lncRNAs categorized based on the number of abstracts retrieved per lncRNA from PubMed. This analysis suggests that the more the number of abstracts available for a lncRNA, the higher is the recall for annotations extracted by Lantern. Our data also shows an optimal peak of recall at 100 to 150 abstracts extracted per lncRNA

LncRNAdb is a resource that was generated by manually curating literature, which enables capturing of deeper functional annotations which contemporary natural language processing approaches cannot retrieve, which is why there is a variation in the number of lncRNAs that are recorded on lncRNAdb and the number of lncRNAs which had PMIDs annotated. Also, our approach extracts annotations that are solely based on the abstract retrieved, as we observed that annotations from other sections of the articles gave rise to substantial noise in the annotations. While extracting the abstracts for respective lncRNA, we observed that the recall spikes as the number of annotated abstracts increases, however the same depletes as the number of abstracts exceeds 150 as seen in Fig. 2. Therefore, we administered an upper limit of 150 abstracts, to reduce the noise or non lncRNA specific abstracts. We put forth the rationale for selecting the four ontologies annotated as gene ontology, human phenotype ontology, disease ontology, and SNOMEDCT because these were the most representative of lncRNA function and are tangible for translational observations. While benchmarking the retrieved ontology annotations, the number of annotations retrieved for human phenotype ontology, disease ontology, and SNOMEDCT were very few from the gold standard to make any comparisons. NCBO returns 5 scores with respect to annotations for each ontology, however, these scores represent the most appropriate annotated ontology for a given text, which in the case of Lantern are auxiliary, as Lantern tries to extract annotations rather than compare ontologies. Therefore, to filter the extracted annotations we employed a frequency of occurrence-based approach. In the post-search result page of the Lantern, annotated ontology IDs are sorted and displayed by their frequency of occurrence across the source. Another filter we employed was filtering out the non-informative, non-specific annotations like “gene”, “cell”, “protein”. This filtration step was performed by manually mining through all the annotations and identifying 1478 non-informative annotations and removing these annotations from the database tables. Lantern also provides additional features and integrated multi-omics resources for the LncRNAs as follows:

#### Mapping lncRNA-RBP interactions by mining publicly available CLIP-seq datasets

Long non-coding RNAs are well known to interact with RNA binding proteins (RBP) in a function-specific and cell-type-specific manner [37]. To understand the functional role of the non-coding transcriptome, it is crucial to dissect and record RBP–lncRNA interaction profiles. Therefore, we extracted lncRNA-RBP interactions from UV cross-linking protocol-based protein-RNA interaction (Crosslinking and Immunoprecipitation;CLIP) experiments from ENCODE [38] project, for 7942 lncRNAs and 139 RBPs in 18 cell lines. The downloaded bed file containing the cell line specific binding site coordinates of 139 RBPs were parsed thoroughly over the annotated lncRNA loci from GENCODE using BEDTools ‘intersect’ option [39]. The extracted 468,348 interactions of RBPs and lncRNAs were tabulated and mounted onto the Lantern database for efficient retrieval and visualization in the form of a network. Additionally, we extracted the Pfam domain information of interacting RBPs from the biomart [40] and incorporated it in Lantern to display the Pfam ID and description along with the cell lines. Also, we further analyzed CLIP-seq based lncRNA-RBP interactions to identify the RBP families most frequently interacting with the long non-coding transcriptome. From our examination, we observed that the RBPs belonging to HNRNP and CSTF2 groups were the most interactive with 5538 lncRNAs associated on average.

#### Comparative visualization of lncRNA expression profiles across human tissues from GTEx RNA-seq samples

To understand how each lncRNA abundance varies across human tissues, we downloaded the transcript-level expression profile as a quantification matrix across 53 tissues from the GTEx data portal [41]. RNA-seq derived transcript abundance data were extracted for each lncRNA. After extracting the expression levels across all the available tissues from the GTEx portal, python package ‘pandas’ and ‘pylab’ [42] was used to generate dynamic boxplots presenting the queried lncRNA expression. This segment of Lantern can help the user to visually interpret the nature of 9982 lncRNAs across multiple tissues.

#### Generating lncRNA: protein co-expression associations by integrating tissue-specific expression profiles

Several studies show the role of LncRNAs in multiple biological processes including transcriptional/ post-transcriptional regulation [43–46]. A majority of them are characterized for their conservation across multiple species [47, 48]. Therefore, it is imperative to infer the potential crosstalk between protein(s) and lncRNAs. To establish such molecular interaction between LncRNAs and proteins, we downloaded the protein level abundance of 9983 proteins from the human proteome map (HPM) [49] and lncRNA expression data from the GTEx portal [41] as described previously. We considered the 14 tissues which are common between the two databases, to deploy the co-expression analysis and established a rank-based association network between protein and lncRNA expression levels using spearman rank correlation [50]. From the co-expression analysis, each instance of protein-lncRNA co-expression was annotated with r-value, p-value, and FDR to gauze the intensity and significance of the interaction. The resulting high confidence (5% FDR) association network with a total of 15,314 nodes and 5,038,058 edges across all 5331 lncRNA was visualized for each lncRNA on Lantern using Cytoscape JavaScript framework [51].

#### Prediction of potential upstream regulatory transcription factors of lncRNAs

LncRNAs are known to express in a tissue-specific manner and hence require an investigation of upstream regulators such as transcription factors genome-wide. We made an in silico attempt to navigate the possible TFs regulating the lncRNAs. First, we downloaded the Position Specific Weight Matrices (PWMs) of ~ 2100 transcription factors from TRANSFAC [52] and MEME [53] databases. Also, we used bedtools [54] to extract the 2 kb upstream + 0.5 kb instream sequence from the TSS (Transcript Start Site) of all lncRNAs (based on lncRNA genomic coordinates from Ensembl hg38.p84) in fasta format. Next, PWMs of these TFs were scanned onto the extracted regulatory region of lncRNAs using Find Individual Motif Occurrences (FIMO) [55] with default parameters. The resulting motif-based binding sites were filtered at a 5% FDR cutoff. A total of 617,074 interactions across 161 transcription factors and 10,727 lncRNAs were identified and mounted onto the Lantern interface.

#### Estimating the protein-coding potential of lncRNAs at genome-scale

The coding potential is a useful metric that helps to differentiate non-coding transcripts from other biotypes. Coding potential can not only help to dissect and elucidate the functionality of lncRNAs but also help identify novel lncRNAs [56]. For computing the coding potential of lncRNAs, we extracted the genomic coordinates of lncRNA transcripts from GENCODE (hg38) in GTF (Gene Transfer Format) and deployed a robust machine learning-based approach, Coding Potential Calculator 2 (CPC2) [57], which considers sequence features to estimate the coding potential of a transcript. CPC2 was deployed across human lncRNA coordinates, acquired in the form of GTF from GENCODE, to generate the coding potential estimates for all lncRNA. The coding potential of 27,907 lncRNA transcripts, mapped to 9898 lncRNAs can be seen on Lantern along with intrinsic information such as peptide length or length of the open reading frame, Fickett score [58] for the transcript, isoelectric point, and open reading frame integrity, which depicts if the ORF starts and stops with the appropriate codons.

#### Annotating and visualizing lncRNAs’ preferential sub-cellular localization

A crucial element that directly affects the function of a given transcript is its cellular localization [59]. Similarly, the function of lncRNAs’ is also influenced by where the lncRNA is localized in the cell [60, 61]. Therefore, it is critical to understand the compartment-specific localization of lncRNA. To investigate this, we obtained the lncRNA localization information from lncATLAS [62], which was originally computed from ENCODE consortium data [38]. The lncRNA expression localization information of around 11 K lncRNAs across 15 cell lines and 10 sub-cellular compartments was processed and mounted onto the database, to be visualized as a heatmap with cell lines and sub-cellular compartments as axes. The heatmap can be viewed in two normalization formats, row normalized (i.e. normalized with respect to compartments across each cell line), which is the default, and column normalized (i.e. normalized with respect to cell lines across each compartment). The heat map visualization was enabled using the high charts JavaScript framework.

#### Transcriptome-wide identification of eQTL and GWAS SNPs occurring on lncRNAs

Another key factor that can influence the functions of lncRNAs in a cellular context is the single nucleotide polymorphism (SNP) [63–65]. It is reported that more than 85% of SNPs that are associated with disease occur in the non-coding regions [66]. This makes it imperative to explore various SNPs associated with lncRNAs. Therefore, to identify SNPs pertaining to lncRNAs, and their effect on the cellular and tissue level behavior, we mapped SNPs from the GWAS catalog and GTEx eQTL analysis onto lncRNAs. The latest version of the GWAS SNP association was obtained from the GWAS catalog [67]. To examine for SNPs across lncRNA, SNP information from GWAS was processed and converted to BED format. Using BEDTools [54], 5897 GWAS SNPs whose coordinates overlapped with lncRNA coordinates, obtained from GENCODE, were extracted along with their physical trait information. Each GWAS SNP on lncRNA is annotated with the dbSNP ‘rsid’ [68], position, and phenotypic trait.

The Expression quantitative trait locus (eQTL) analysis from the GTEx project [41] has mapped SNPs to altered gene expression across 48 tissues. This expression dysregulating tissue-specific 39,741 SNPs were mapped onto lncRNAs. The obtained eQTLs were recorded along with information such as dbSNP ‘rsid’ for the respective SNP, tissue in which the SNP was observed, genomic coordinates, and an r-value. Thereby recording 45,530 SNPs in 6714 lncRNAs, across 48 tissues, along with GWAS annotated phenotypic trait and GTEx annotated eQTL information. This extensive SNP-lncRNA annotation can help understand and explore the phenotypic nature of SNPs across lncRNAs.

### Database and web interface

A web resource was developed for hosting all the extracted and benchmarked annotations. In the back-end, a MySQL database was used to create and connect tables with lncRNA-specific annotations. The database schema was a straightforward connection across each annotation component as a separate table and lncRNA names as a unique table, associated with the lncRNA name. The front-end is a ‘php’ based, Twitter bootstrapped webpage, developed to generate a user-friendly and intuitive interface. The search boxes are enabled with auto-suggestions based on user input using jQuery.

Lantern can be browsed for annotations via two search routes, user can search by lncRNA, using lncRNA names and Ensembl gene ID (ENSG), by toggling between search buttons above the search bar, users can also search for ontologies on the adjacent search tab, to retrieve all the lncRNAs annotated to that ontology. Lantern also provides network visualizations of lncRNA and its annotations across ontology annotation, lncRNA-RBP interactions, and protein co-expression. These network visualizations were enabled using the Cytoscape JavaScript framework. All the tables retrieved after search on Lantern can be downloaded. Some columns in the tables retrieved after search on Lantern are hyperlinked to various resources, showing additional information about that respective tuple. For instance, the PMID column in the ontology annotation retrieved table redirects the user to all the literature that supports the annotation. Similarly, the ontology ID column, gene ID columns, Pfam ID column, and dbSNP rsid columns are as well hyperlinked. In the tissue-specific lncRNA expression section, we embedded a hyperlink to FireBrowse [69] which redirects the user to the specific lncRNA’s expression across multiple cancers. The tissue-specific expression of lncRNA is depicted as boxplots across the tissues, this image can be downloaded using the embedded buttons. The sub-cellular localization of lncRNA can be visualized as a heatmap showing intensity of a lncRNA’s abundance across compartments and cell lines, this image can be exported in multiple formats. The downloads page on Lantern enables the user to retrieve and download all available information for that lncRNA on one page. The documentation page briefly describes the implementations on Lantern and the user guide page enables users to get an overview of all the functionalities on Lantern.

### Utility and discussion

Lantern facilitates analysis and annotation of lncRNAs on multiple levels using established computational pipelines. It employs a swift integration of Application Programming Interface (API), established tools, and resources to extract the most recent and relevant lncRNA functional annotations. The ontology annotations available on Lantern were extracted by a semi-automated pipeline which leverages the potential of Natural Language Processing (NLP), by using it to mine recent lncRNA literature. The extracted ontology annotations are benchmarked against manually curated gold standard lncRNA annotation resources. Lantern also houses lncRNA annotations across a range of omics data, which provides extensive insights into lncRNA’s role from a fundamental level, as cellular functions, to an empirical scale such as tissue level. Information such as a list of RBPs that have been experimentally identified to interact with lncRNAs, proteins that co-express with lncRNAs, tissues that are more representative of a specific set of lncRNAs, sub-cellular expression of lncRNAs, and Single Nucleotide Polymorphisms in lncRNAs can essentially enable the exploration of the functional role of lncRNAs. Lantern attempts to bridge this knowledge gap with accurate and up-to-date lncRNA annotations.

#### Utility case

**Fig. 3:**
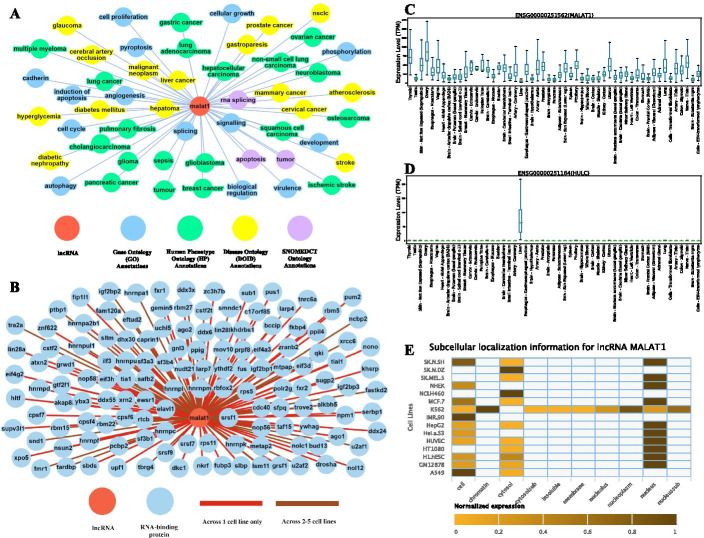
Selection of multi-level genomic and ontology annotations for lncRNA MALAT1 as seen on Lantern. **A** ontology annotation interaction network showing accurate ontology annotations of lncRNA MALAT1, extracted from literature mining. **B** RNA binding protein interaction profile of MALAT1 across multiple cell lines. **C** Boxplot showing global expression of lncRNA MALAT1 across tissues. **D** Boxplot showing liver-specific expression of lncRNA HULC indicative of upregulation in liver tissue, emphasizing Lantern's ability to capture tissue-specific expression profile of lncRNAs. **E** Heatmap showing the sub-cellular localization of MALAT1 in various compartments across multiple tissues. In agreement with established observations, MALAT1 was found to exhibit enriched abundance in the nucleus over cytosol

LncRNAs have been observed to be involved in a spectrum of regulatory processes, including remodeling of chromatin, regulation of splicing, translation of mRNA, and stability of mRNA, and protein [70]. Despite having a broad range of regulatory roles, lncRNAs are known to have tissue or phenotype exclusive regulatory facets [42, 47, 71, 72]. Thereby it is important to, not only understand lncRNAs and cellular elements interacting with them but also to investigate the role of lncRNAs in the context of tissue or phenotype exclusivity. Lantern precisely captures such annotations of lncRNAs and projects them via dynamic visualizations. For instance, metastasis-associated lung adenocarcinoma transcript 1 (MALAT1) is one of the extensively studied lncRNAs. It is known to play a key role in various disorders like lung adenocarcinoma [73–76], hepatocellular carcinoma [77], ovarian cancer [78], osteosarcoma [73, 79], and atherosclerosis [80], all of which Lantern captures and portrays as an ontology network as seen in Fig. 3A. MALAT1 is observed to be upregulated in various types of cancer and acts as a proto-oncogene in hepatocellular carcinoma [81, 82]. It is widely known to interact with the oncogenic splicing factor SRSF1[83]. Lantern captures the phenotypic annotations attributed to hepatocellular carcinoma that were documented previously along with its known tissue-specific biological interaction with SRSF1 observed in HepG2 [84]. In addition to that, Lantern also provides other accurately annotated RBP interactions as shown in Fig. 3B. These interactions highlight the underlying mechanism of RNA binding proteins and lncRNAs as co-mediators of cellular functions and phenotypes, as previously seen in literature [85–89]. Therefore, such interactions available on Lantern facilitates research groups to further understand the potential lncRNA-centric regulome and associated co-complexing RBPs.

To further portray and understand lncRNAs’ expression pattern in multiple tissues, we downloaded the processed expression profile of lncRNAs across 53 tissues from the GTEx portal [41]. We integrated the dataset in Lantern as a boxplot to show the comparative expression plot that helps the user to visually interpret the nature of 9982 lncRNAs. MALAT1, as described previously is observed with a global expression profile across multiple tissues, as seen in Fig. 3C, in coherence with the range of phenotypes and diseases it is associated with. In contrast, Lantern also reports certain highly tissue-specific lncRNAs. For instance, lncRNA HULC, which is known to be highly expressed in hepatocellular carcinoma [90–93], demonstrates a distinct signal of upregulation in the liver, as seen in Fig. 3D.

LncRNAs are recruited based on their functional role and abundance in a given sub-cellular compartment [94, 95]. Therefore, it is essential to understand and explore the respective sub-cellular compartment abundance of lncRNAs. Lantern provides an interactive visual platform to investigate the lncRNA abundance across cellular compartments in 15 cell lines, available as a heatmap. MALAT1 for instance has extensively been identified as a nuclear lncRNA [96–99].In agreement with previous literature [84], localization of MALAT1 was observed predominantly in the nuclear compartment of the cell (Fig. 3E).

**Fig. 4:**
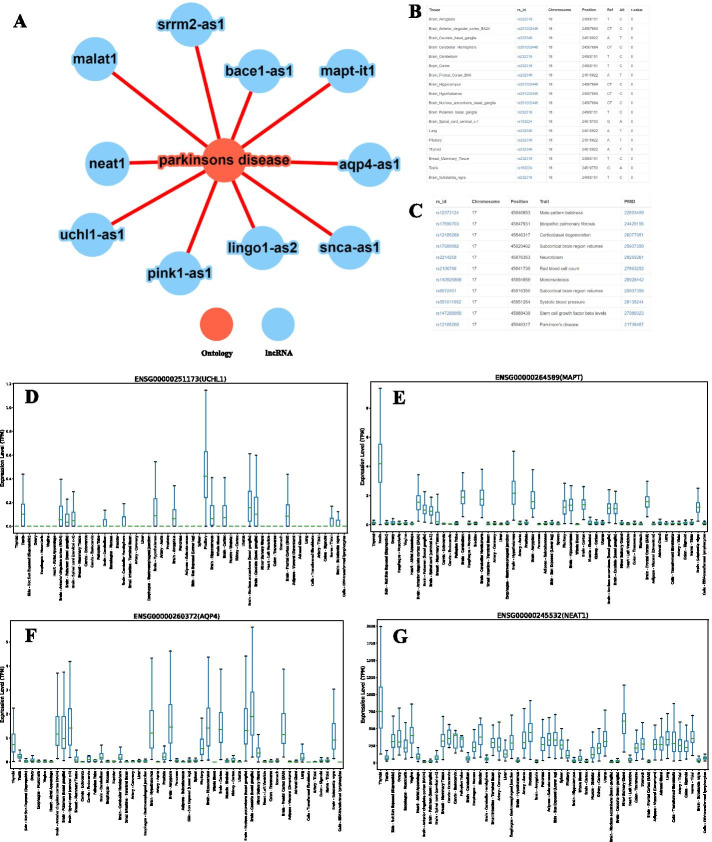
lncRNAs annotated to Parkinson’s disease and their pertaining information as seen on Lantern. **A** Network depicting the lncRNAs associated with the ontology ‘Parkinson’s disease’, illustrating the preciseness of annotations. **B** Screenshot of tissue-specific SNPs in lncRNA AQP4-AS1 from GTEx eQTL. An enriched abundance of SNPs in brain tissue reinforcing the association of AQP4-AS1 and Parkinson’s disease. **C** Screenshot of GWAS SNP phenotype annotations for lncRNA MAPT-IT1 showing its established association with Parkinson’s disease, corroborating Lantern’s annotations. **D**–**G** boxplots showing brain-exclusive varied expression of lncRNAs annotated to Parkinson’s disease as seen on Lantern. Panel **D**, **E**, and **f** showing elevated expression levels in brain tissue for UCHL1-AS1, MAPT-IT1, and AQP4-AS1 respectively. While in panel **G** lncRNA NEAT1 with exclusive modest abundance across all brain tissues supporting its established association as identified by Lantern

Lantern also provides lncRNA disease phenotype associations. Our understanding of a disease phenotype can improve by recognizing the various cellular elements involved. To identify and understand lncRNAs’ functional role in various disease phenotypes, an ontology-based search was integrated onto Lantern. Users can search for a phenotype and Lantern will return all the associated lncRNAs, based on the functional annotations extracted from the literature by the Mobius pipeline (see “Construction and Content” section). A sample phenotype-centric network is visualized as Fig. 4A, wherein the human disease ontology ‘Parkinson’s disease has been annotated with several lncRNAs by the Mobius pipeline, including BACE1-AS1[100], MAPT-IT1[101, 102], AQP4-AS1[103] and SNCA-AS1[104], etc., along with the peer-reviewed literature from which this phenotype-lncRNA association was extracted. Lantern also integrates phenotype-specific SNPs across lncRNAs. Reinforcing the annotations for lncRNAs like AQP4-AS1 and MAPT-IT1, which were annotated to ‘Parkinson’s disease by the Mobius pipeline, the tissue-specific SNPs in AQP4-AS1 predominantly occur in brain tissue and GWAS SNP annotation for MAPT-IT1 can be seen as ‘Parkinson’s Disease’ from Fig. 4B, C.

As evident from Lantern’s tissue-specific expression panel, these lncRNAs viz. UCHL-AS1, MAPT-IT1, and AQP4-AS1 were observed to be relatively elevated in brain tissue (Fig. 4D–F), signifying their regulatory role in the normal functioning of the brain, in coherence with the previously reported observations [105–107]. In contrast, lncRNA NEAT1 was found to be down-regulated across all the GTEx brain regions (Fig. 4E), which corroborates with our current understanding that NEAT1 has been extensively observed to be upregulated in the brain and neurodegenerative disorders [108, 109]. Since the expression on Lantern is extracted from GTEx based normal tissue abundance, which is why NEAT1 expression across normal brain tissue is highly downregulated.

**Fig. 5:**
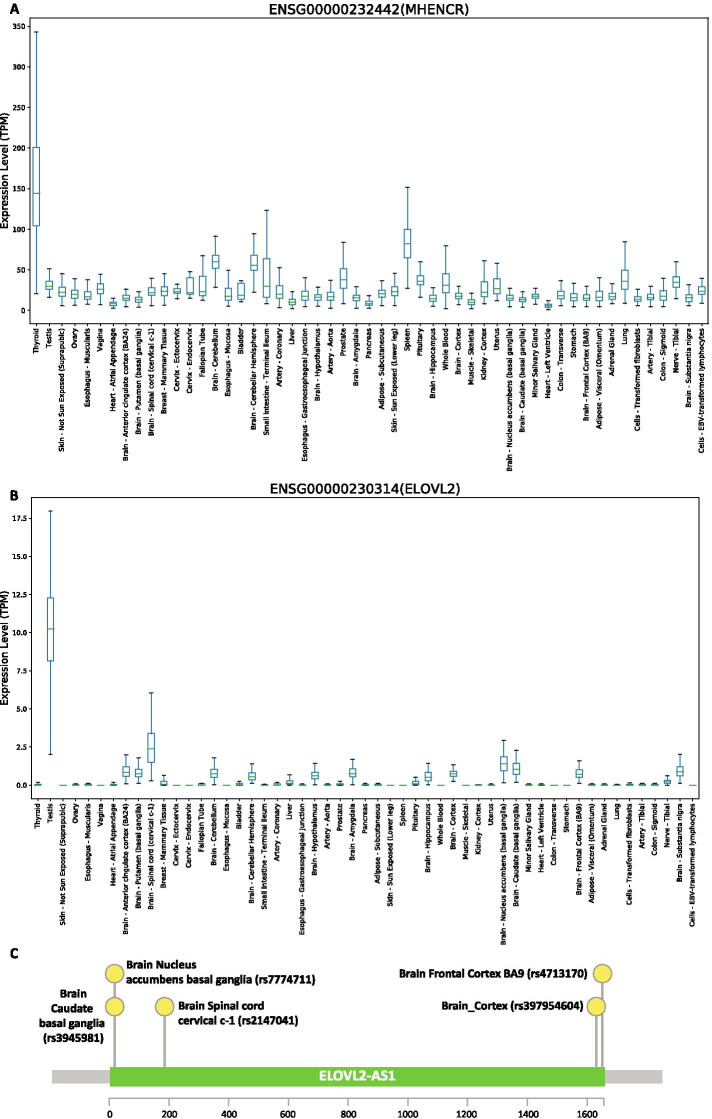
Understanding the functional role of novel and not extensively studied lncRNAs. **A** Plot showing tissue-specific expression of lncRNA MHENCR. **B** Tissue-specific expression plot showing brain-specific expression for novel lncRNA ELOVL2-AS1. **C** Lollipop plot showing the predominant occurrence of ELOVL2-AS1 SNPs in the brain

Lantern can not only help explore the regulatory role of extensively studied lncRNAs but can also recognize novel associations and features of non-putative lncRNAs. LncRNAs have been widely recognized for varying functions across different tissues and phenotypes.For instance lncRNA XISTis recognized as an oncogene in human colorectal cancer [110] but also acts as a tumor suppressor in prostate cancer [111]. Increasing evidence for the variable role of lncRNAs generates the necessity for an understanding of the novel as well as well-studied lncRNAs in a cell line or tissue-specific manner. For instance, the functional role of lncRNA MHENCR is not extensively explored. Although, it is known to be upregulated in melanoma [112] and is implicated with a poor survival rate in melanoma patients. This oncogene MHENCR is known to be influence cell proliferation and apoptosis by attenuating miR-425 and miR-489, thus upregulating their target genes IGF1, SPIN1, and activating the PI3K-Akt pathway. In corroboration to this evidence, MHENCR expression is recorded to be low in GTEx extracted tissue expression. However, we observed a 30-fold upregulation of MHENCR expression in thyroid tissue, as seen in Fig. 5A. The role of MHENCR has not been elucidated previously in the context of thyroid or related phenotypes. This upregulation of this lncRNA in the thyroid could potentially mean that it is essential for the normal function of the thyroid. Interestingly, we also observed that a GTEx eQTL analysis-based SNP was also identified in the locus of this lncRNA.

With these observations, Lantern not only provides a platform to recognize the role of lncRNA in multiple diseases but can also facilitate the identification of tissue-specific intrinsic features of lncRNA which is indispensable for understanding their molecular functions. Additionally, annotations from resources like TRlnc [113] combined with ontology and omic-annotations from Lantern can enable us to develop a better understanding of transcriptional regulatory mechanisms of lncRNAs.

Another interesting novel lncRNA was ELOVL2-AS1, unlike its protein-coding counterpart ELOVL2-AS1 which is not fully studied. However, Lantern registers its interactions with various RNA binding and cellular proteins like SRSF1, RBFOX2, and PTBP1 which have a significant role in cellular processes like splicing and development especially in the context of the brain [114]. We observe a specific expression pattern of ELOVL2-AS1 from Lantern, even though it has higher expression in testis, from Fig. 5B, we observed that ELOVL2-AS1 has a relatively higher abundance across all of the brain tissues. Moreover, we also observed that 5 out of 6 SNPs (rs3945981, rs397954604, rs4713170, rs7774711, rs2147041) annotated by GTEx eQTL analysis, identified in and around its locus were predominantly from brain tissues, seen in Fig. 5C. With such extensive multi-level lncRNA annotations, Lantern has the potential to aid the identification of several other novel lncRNAs, as well as facilitate and develop a hypothesis around well-studied lncRNAs to understand their regulatory role in diseases.

## Conclusion

This study describes the development of a semi-automated pipeline for annotating lncRNA with ontology information using literature mining. The extracted ontology annotations were benchmarked against a manually curated lncRNA information resource. All the extracted ontology information is deposited onto a web interface to easily navigate and retrieve ontology annotations for lncRNA. A molecular-level interaction profile of lncRNAs was put together, along with tissue-specific and SNP information utilizing contemporary NGS data analysis pipelines. Subsequently, a public resource with high-quality-controlled ontology annotations and comprehensive omics annotations was developed for improving the annotation of the non-coding transcriptome.

## Supplementary Information

**Additional file 1: Table S1.** Manual precision calculation for benchmarking extracted ontology annotations.

## Data Availability

Lantern database is hosted at sysbio.lab.iupui.edu/lantern, which provides downloadable tables and figures. Source codes are available at GitHub (at https://github.com/VidhurDS/Lantern) [115].

## Abbreviations

LncRNAs: Long non-coding RNAs
SNP: Single nucleotide polymorphism
GTEx: Genotype-tissue expression
GWAS: Genome-wide association studies
RBP: RNA binding protein
CLIP: Crosslinking and immunoprecipitation
NLP: Natural Language Processing
GO: Gene ontology
HPO: Human phenotype ontology
DO: Disease ontology
SNOMEDCT: Systematized nomenclature of medicine—clinical terms
NGS: Next-generation sequencing
API: Application programming interface
NCBO: National Center for Biomedical Ontology
HPM: Human proteome map
TSS: Transcript start site
FIMO: Find Individual Motif Occurrences
GTF: Gene transfer format
ORF: Open reading frame
ENSG: Ensembl gene ID
